# New‐onset and persistent neurological and psychiatric sequelae of COVID‐19 compared to influenza: A retrospective cohort study in a large New York City healthcare network

**DOI:** 10.1002/mpr.1914

**Published:** 2022-06-15

**Authors:** Andrei L. Iosifescu, Wouter S. Hoogenboom, Alexandra J. Buczek, Roman Fleysher, Tim Q. Duong

**Affiliations:** ^1^ Department of Radiology Albert Einstein College of Medicine and Montefiore Medical Center Bronx New York USA

**Keywords:** COVID‐19, influenza, neuropsychiatry, new‐onset symptoms

## Abstract

**Objectives:**

Neurological and neuropsychiatric manifestations of post‐acute SARS‐CoV‐2 infection (neuro‐PASC) are common among COVID‐19 survivors, but it is unknown how neuro‐PASC differs from influenza‐related neuro‐sequelae. This study investigated the clinical characteristics of COVID‐19 patients with and without new‐onset neuro‐PASC, and of flu patients with similar symptoms.

**Methods:**

We retrospectively screened 18,811 COVID‐19 patients and 5772 flu patients between January 2020 and June 2021 for the presence of new‐onset neuro‐sequelae that persisted at least 2 weeks past the date of COVID‐19 or flu diagnosis.

**Results:**

We observed 388 COVID‐19 patients with neuro‐PASC versus 149 flu patients with neuro‐sequelae. Common neuro‐PASC symptoms were anxiety (30%), depression (27%), dizziness (22%), altered mental status (17%), chronic headaches (17%), and nausea (11%). The average time to neuro‐PASC onset was 138 days, with hospitalized patients reporting earlier onset than non‐hospitalized patients. Neuro‐PASC was associated with female sex and older age (*p* < 0.05), but not race, ethnicity, most comorbidities, or COVID‐19 disease severity (*p* > 0.05). Compared to flu patients, COVID‐19 patients were older, exhibited higher incidence of altered mental status, developed symptoms more quickly, and were prescribed psychiatric drugs more often (*p* < 0.05).

**Conclusions:**

This study provides additional insights into neuro‐PASC risk factors and differentiates between post‐COVID‐19 and post‐flu neuro‐sequelae.

## 1 INTRODUCTION

Many survivors of coronavirus disease 2019 (COVID‐19) experience lingering neurological or neuropsychiatric symptoms that can persist for many months (Davis et al., 2021; Rubin, 2020; Zhao et al., 2020). These symptoms, often referred to as neurological post‐acute sequelae of SARS‐CoV‐2 infection (neuro‐PASC), include, but are not limited to, altered mental status (AMS), altered taste and smell, anxiety, ataxia, delirium, depression, dizziness, fatigue, headaches, memory loss, nausea, new‐onset post‐traumatic stress disorder, seizures, strokes, and tinnitus (Collantes et al., 2021; Graham et al., 2021; Moghimi et al., 2021; Wang et al., 2020). Neuro‐PASC may be caused by an immune response to initial infection, by direct viral infection of the central nervous system, or by psychological stressors such as social isolation, fear of illness, stigma, and future uncertainty (Anand, 2021, Mazza et al., 2020). Neuro‐PASC symptoms affect individuals of all age groups, including young children (Ludvigsson, 2021). Emerging data suggests that individuals with mild symptoms from SARS‐CoV‐2 infection (i.e., not requiring hospitalization) are also susceptible (Townsend et al., 2020; van den Borst et al., 2021).

Neuro‐PASC is incompletely understood, and prior studies have largely relied on subjective survey reports, often without detailed clinical data. Moreover, it is unknown whether neuro‐PASC symptoms differ from neurological and neuropsychiatric symptoms associated with other respiratory diseases such as influenza, and to what extent these neuro‐PASC symptoms are new‐onset and persistent in nature.

### 1.1 Aims of the study

The aim of this study was to characterize neuro‐PASC in COVID‐19 patients without prior history of neurological or neuropsychiatric symptoms using detailed real‐world data pre‐ and post‐SARS‐CoV‐2 infection, and to compare with both COVID‐19 patients without neuro‐PASC and influenza patients with neurological or neuropsychiatric sequelae from the same catchment area and study timeframe. We evaluated clinical and demographic patient variables, neuro‐PASC symptoms, timeframes of symptom onset, and patient drug prescriptions associated with neuro‐PASC.

## 2 MATERIAL AND METHODS

This study was approved by the Einstein‐Montefiore Institutional Review Board with an exemption for informed consent and a HIPAA waiver due to the retrospective and deidentified nature of the data.

### 2.1 Data source

All data originated from observational databases in the Montefiore Health System as described in previous reports (Hoogenboom, Fleysher, et al., 2021; Hoogenboom, Pham, et al., 2021; Lu, 2021; Lu et al., 2022). Briefly, patient data between January 1, 2020, and June 9, 2021, were standardized to the Observational Medical Outcomes Partnership (OMOP) Common Data Model (CDM) version 6, which uses standard vocabulary concepts, allowing for the systematic analysis of disparate observational databases such as electronic medical records and disease classification systems (e.g., ICD‐10, SNOWMED, LOINC) (Hripcsak et al., 2015). Cohort building and searches of vocabulary concepts were performed using ATLAS, a web‐based tool developed by the Observational Health Data Sciences and Informatics (OHDSI) community that enables navigation of patient‐level, observational data in the CDM format. OMOP concept IDs used to define neuro‐PASC symptoms are presented in Table S1. Data was subsequently imported into an SQLite database (www.sqlite.org) and queried using the DB Browser (version 3.12.2).

### 2.2 Study population and inclusion criteria

All individuals included in this report were patients of the Montefiore Health System, one of the largest healthcare systems in New York City with more than 180 primary and specialty care locations in the Bronx environs serving a large racially and ethnically diverse population (Hoogenboom, Pham, et al., 2021; Wadhera et al., 2020). Three groups of patients were studied: (1) COVID‐19 patients with neuro‐PASC symptoms, (2) COVID‐19 patients without neuro‐PASC symptoms, and (3) Flu patients with neurological and neuropsychiatric symptoms.

For the COVID‐19 cohort, all individuals who tested positive for SARS‐CoV‐2 infection (laboratory confirmed by real‐time PCR test) were included. Patients were subsequently screened for the presence of new‐onset neurological and neuropsychiatric symptoms that persisted at least 2 weeks past the date of COVID‐19 diagnosis, the standard period during which SARS‐CoV‐2 has been found to replicate (Lamontagne et al., 2021), to exclude transient, hyperacute effects.

Patients were included in the flu cohort if diagnosed with influenza (by probe detection of Influenza Virus A or B RNA) in the same hospital system and within the same timeframe, but without a positive COVID‐19 diagnosis.

### 2.3 Demographics and clinical variables

Collected data included demographics, comorbidities, and vital signs. Demographic data included age, sex, race, and ethnicity. Race/ethnicity were based on patient self‐identification and categorized as American Indian, Asian, Black, Pacific Islander, White, or Other (comprising patients selecting multiple groups or some other race). Chronic comorbidities included hypertension, diabetes, congestive heart failure (CHF), chronic kidney disease (CKD), coronary artery disease (CAD), chronic obstructive pulmonary disease (COPD), and asthma. COVID‐19 disease severity was defined by hospitalization status (i.e., outpatient vs. inpatient general floor or ICU), which is indicative of need for escalated care. Vitals and laboratory values were collected at admission and included body mass index (BMI), pulse oximetry, oral temperature, lactate dehydrogenase (LDH), C‐reactive protein (CRP), D‐dimer, blood urea nitrogen (BUN), lymphocytes, and leukocyte measurements.

### 2.4 Statistical analysis

Statistical analyses were performed using R programming language (version 4.0.2), RStudio (version 1.3.1056), Microsoft Excel for Mac (version 16.52), and Excel add‐in program Analysis ToolPak. We used descriptive statistics to report patient demographic characteristics, including mean ± standard deviation (e.g., age, BMI, pulse oximetry). Sex, race, ethnicity, comorbidities, and hospitalization were reported as *n* (% of cohort). All *t*‐tests, chi‐square tests, analysis of variance (ANOVA) analyses, and logistic regression analyses were evaluated at a significance level of *α* < 0.05, adjusted for multiple comparisons using Bonferroni corrections.

## 3 RESULTS

### 3.1 Demographics, vitals, and laboratory values

**FIGURE 1:**
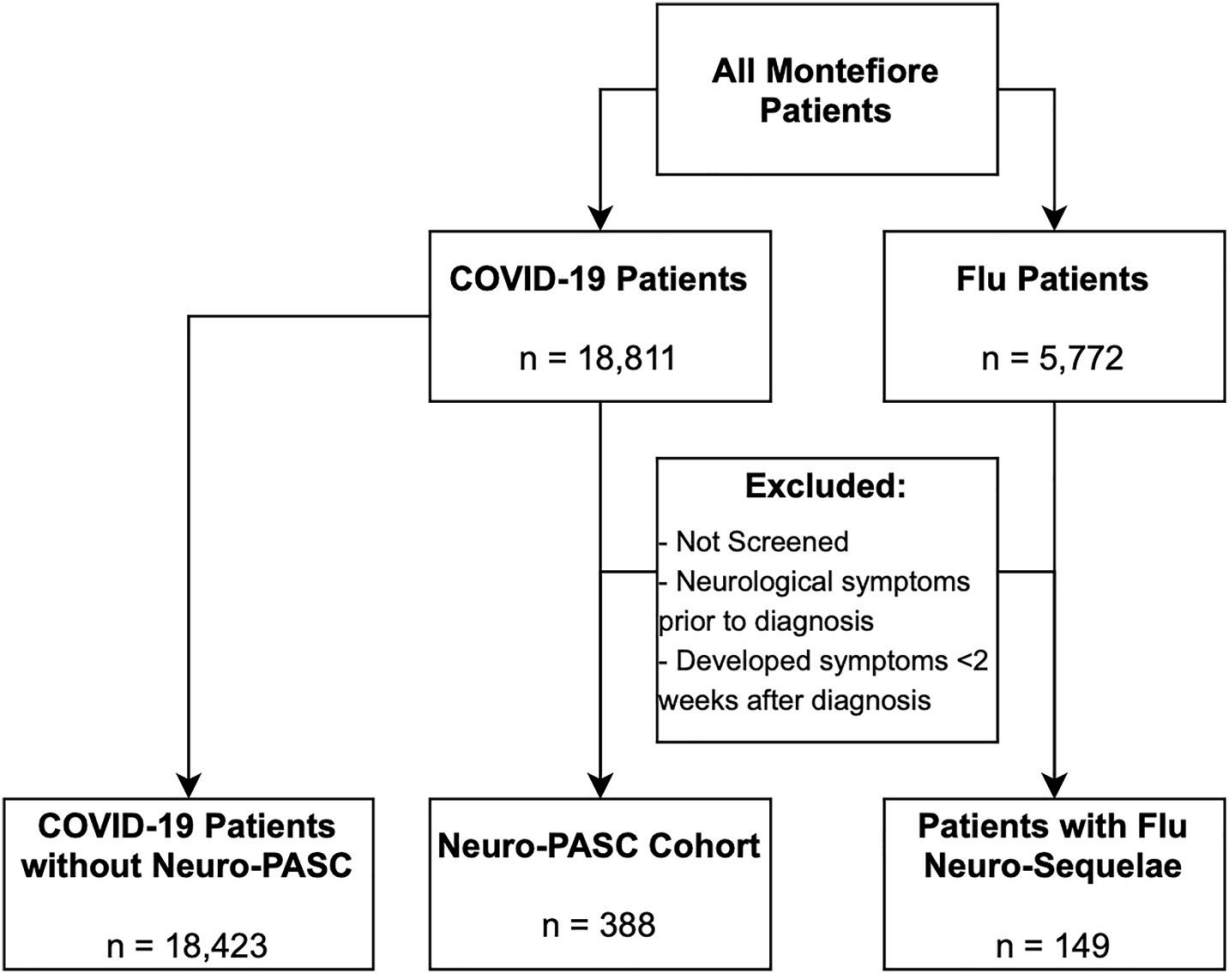
Inclusion/exclusion criteria for neuro‐PASC and control cohorts

Figure 1 displays the COVID‐19 and flu patient selection flowchart. Between January 1, 2020, and June 9, 2021, there were 388 COVID‐19 patients who reported new‐onset neuro‐PASC at least 2 weeks after COVID‐19 diagnosis versus 18,423 COVID‐19 patients without neuro‐PASC. Over the same period, there were 149 flu patients who reported new‐onset of neurological and neuropsychiatric symptoms at least 2 weeks after flu diagnosis.

Table 1 presents demographic and clinical characteristics of the study cohorts. Compared to COVID‐19 patients without neuro‐PASC, COVID‐19 patients with neuro‐PASC symptoms were on average 2 years older (*p* = 0.040), more likely female (63.7% vs. 51.0%, *p* < 0.001), and had lower LDH (*p* < 0.001), CRP (*p* = 0.012) and D‐dimer (*p* < 0.001) measurements. Race, ethnicity, hospitalization status, BMI, pulse oximetry, oral temperature, BUN, lymphocytes, leukocytes, and comorbidities were not significantly associated with neuro‐PASC symptoms (*p* > 0.05).

**TABLE 1:** Demographics, comorbidities, and laboratory data at admission of COVID‐19 patients with and without neuro‐PASC, and flu patients with neurological and neuropsychiatric symptoms

Patient characteristics	COVID‐19 patients with neuro‐PASC *n* = 388	COVID‐19 patients without neuro‐PASC *n* = 18,423	Flu patients with neurological or neuropsychiatric symptoms *n* = 149
Age (years), mean ± SD	57.2 ± 19.3^a^ ^b^	55.2 ± 20.8	40.0 ± 24.0
Female sex, *n* (%)	247 (63.7%)^a^ ^b^	9397 (51.0%)	111 (74.5%)
Race, *n* (%)			
White	47 (12.1%)	2100 (11.4%)	7 (4.7%)
Black	128 (33.0%)	5307 (28.8%)	56 (37.6%)
Asian	13 (3.4%)	628 (3.4%)	3 (2.0%)
Other	175 (45.1%)	7798 (42.3%)	74 (49.7%)
No data	25 (6.4%)	2617 (14.2%)	9 (6.0%)
Ethnicity, *n* (%)			
Hispanic	163 (42.0%)	7208 (39.1%)	83 (55.7%)
Non‐Hispanic	188 (48.5%)	8380 (45.5%)	59 (39.6%)
No data	37 (9.5%)	2907 (15.8%)	7 (4.7%)
Comorbidities, *n* (%)			
Chronic kidney disease	22 (5.7%)	849 (4.6%)	9 (6.0%)
Chronic obstructive pulmonary disease/Asthma	31 (8.0%)^b^	1394 (4.6%)	32 (21.5%)
Coronary artery disease	2 (0.5%)	49 (0.3%)	2 (1.3%)
Diabetes	89 (22.9%)	3579 (19.4%)	33 (22.1%)
Heart failure	23 (5.9%)	887 (4.8%)	7 (4.7%)
Hypertension	104 (26.8%)	3629 (19.7%)	36 (24.2%)
Hospitalization status, *n* (%)			
Outpatient	189 (48.7%)^b^	7471 (40.6%)	124 (83.2%)
Inpatient	169 (43.6%)^b^	9717 (52.7%)	19 (12.8%)
No data	30 (7.7%)	1235 (6.7%)	6 (4.0%)
Vitals and laboratory values, mean ± SD			
Pulse oximetry (%)	88.9 ± 17.3^b^	88.6 ± 16.5	93.8 ± 9.8
Body mass index	30.3 ± 10.8^b^	29.5 ± 8.8	26.5 ± 7.6
Oral temperature (°F)	98.8 ± 1.1	98.8 ± 1.4	98.7 ± 0.7
Lactate dehydrogenase (U/L)	320.0 ± 168.2^a^	380.8 ± 375.3	271.6 ± 92.8
C‐reactive protein (mg/dl)	8.5 ± 9.1^a^ ^b^	10.2 ± 9.9	1.9 ± 2.3
D‐dimer (µg/dl)	2.2 ± 3.1^a^ ^b^	3.2 ± 4.9	0.8 ± 0.8
Blood urea nitrogen (mg/dl)	24.0 ± 21.5^b^	26.4 ± 26.5	14.7 ± 10.2
Lymphocytes (×10^9^/L)	1.6 ± 3.4^b^	1.3 ± 3.0	2.1 ± 1.1
Leukocytes (×10^9^/L)	8.1 ± 5.8	8.1 ± 6.4	8.0 ± 2.4

^a^ Indicates a significant difference between COVID‐19 patients with and without neuro‐PASC symptoms.

^b^ Indicates a significant difference between COVID‐19 patients with neuro‐PASC and flu patients with neurological and neuropsychiatric symptoms.

Compared to flu patients with new‐onset neurological and neuropsychiatric symptoms, the neuro‐PASC COVID‐19 cohort was significantly older (57.2 ± 19.3 vs. 40.0 ± 24.0 years, *p* < 0.001), had fewer females (63.7% vs. 74.5%, *p* = 0.017), and higher hospitalization rate (43.6% vs. 12.8%, *p* < 0.001), BMI (*p* = 0.00068), CRP (*p* < 0.001), D‐dimer (*p* < 0.001), and BUN measurements (*p* < 0.001), but had lower oxygenation levels (*p* < 0.001), lymphocyte counts (*p* = 0.022), and COPD/asthma incidence (*p* < 0.001). There were no group differences in oral temperature, leukocytes, race, ethnicity, or other comorbidities (*p* > 0.05).

### 3.2 Neuro‐PASC symptoms

**FIGURE 2:**
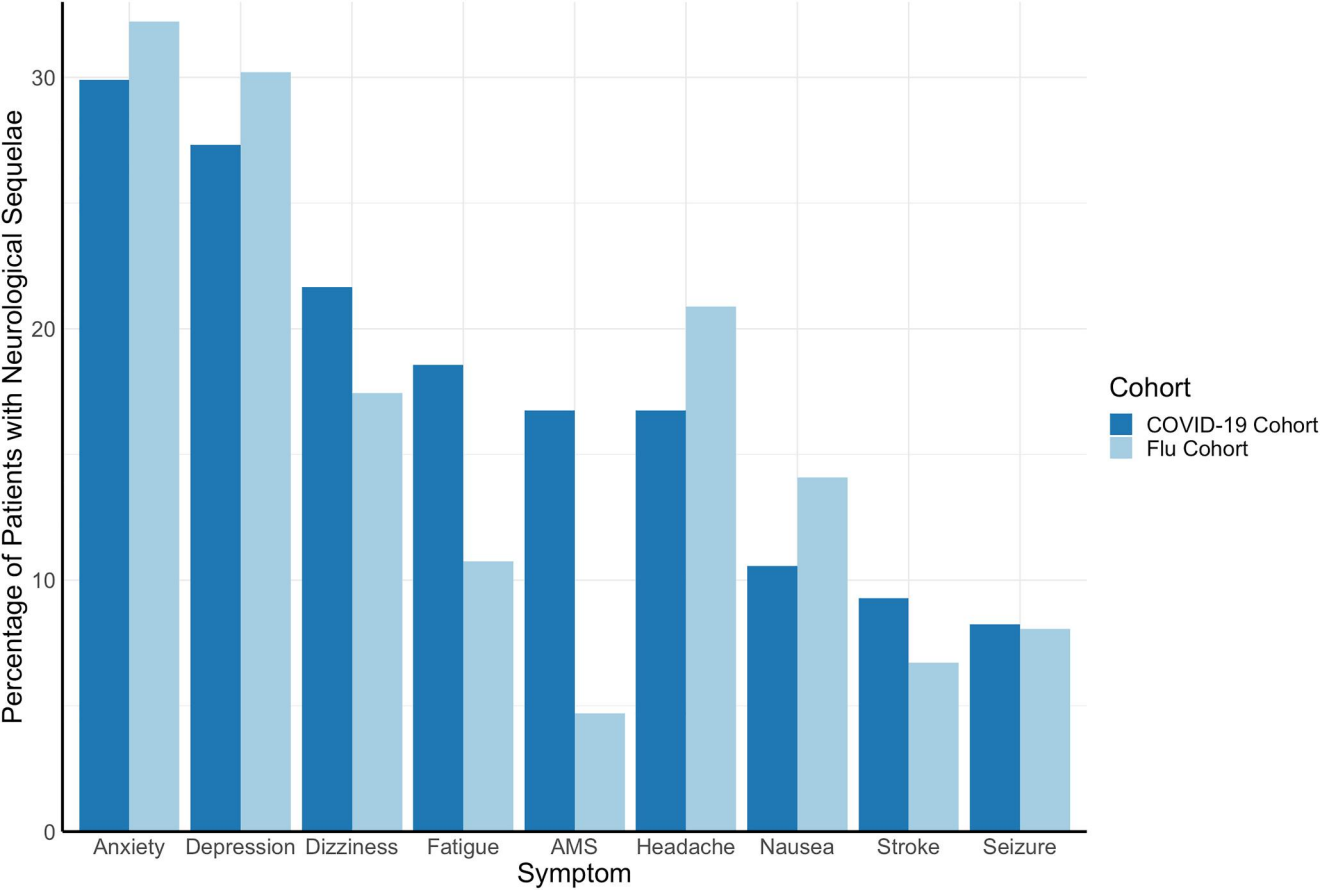
Most prevalent neurological sequelae of COVID‐19 and flu. More COVID‐19 patients experienced altered mental status (AMS) than flu patients (*p* = 0.00014). No significant group differences were observed for any other symptoms

The most common neuro‐PASC symptoms in the COVID‐19 cohort were anxiety disorders (30% of neuro‐PASC patients), followed by depression (27%), dizziness (22%), altered mental status (17%), chronic headaches (17%), and nausea (11%; Figure 2). Similar symptoms were also observed in the flu cohort, except that flu patients experienced lower incidence of altered mental status (*p* = 0.00014).

The average onset of neuro‐PASC symptoms was 138 days post COVID‐19 diagnosis (median = 101 days; Figure 3a), averaging 120 days for inpatients (i.e., ICU or general floor) and 146 days for outpatients (*p* = 0.025, Figure 3b). Age, sex, race, and ethnicity were not associated with time to neuro‐PASC symptom onset (*p* > 0.05). Flu patients displayed significantly more delayed symptom onset, averaging 238 days (*p* < 0.05; Figure 3c).

FIGURE 3(a) Histogram of neuro‐PASC symptom onset timepoints as days from COVID‐19 diagnosis. Red dotted line = average time of symptom onset. (b) Patient hospitalization status versus days to symptom onset (*p* = 0.025). (c) Histogram of neuropsychiatric symptom onset timepoints as days from flu diagnosis

**Figure:**
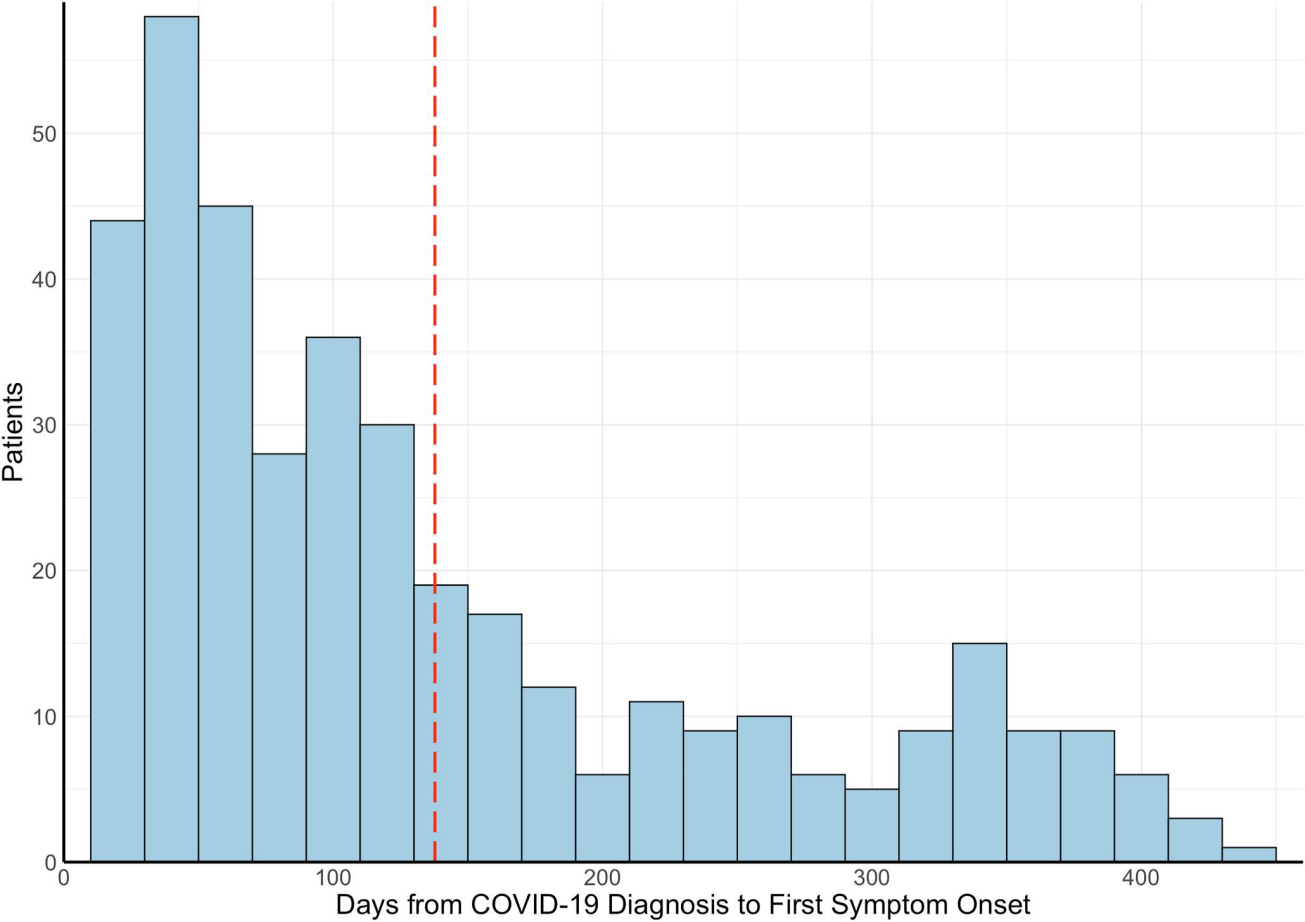

**Figure:**
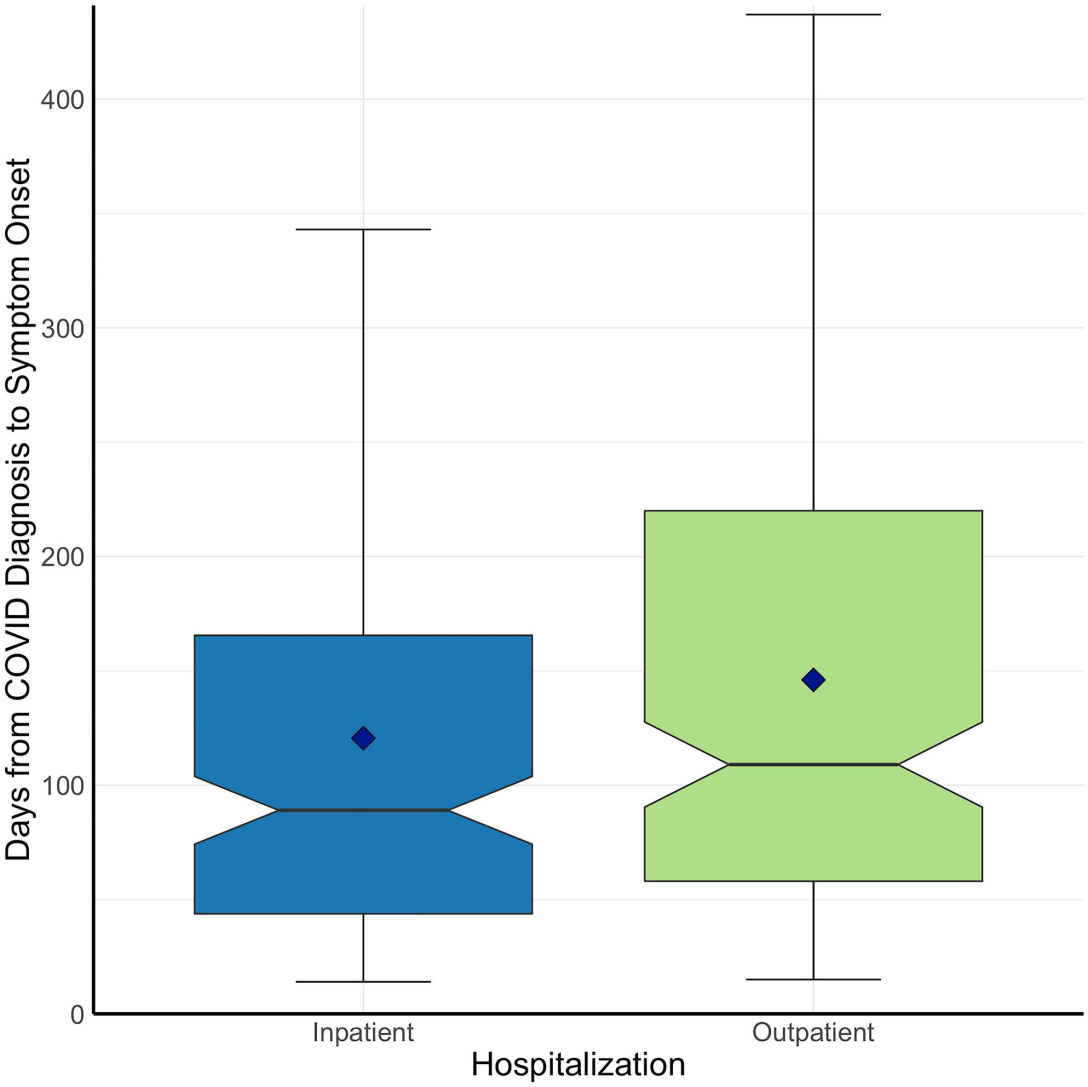

**Figure:**
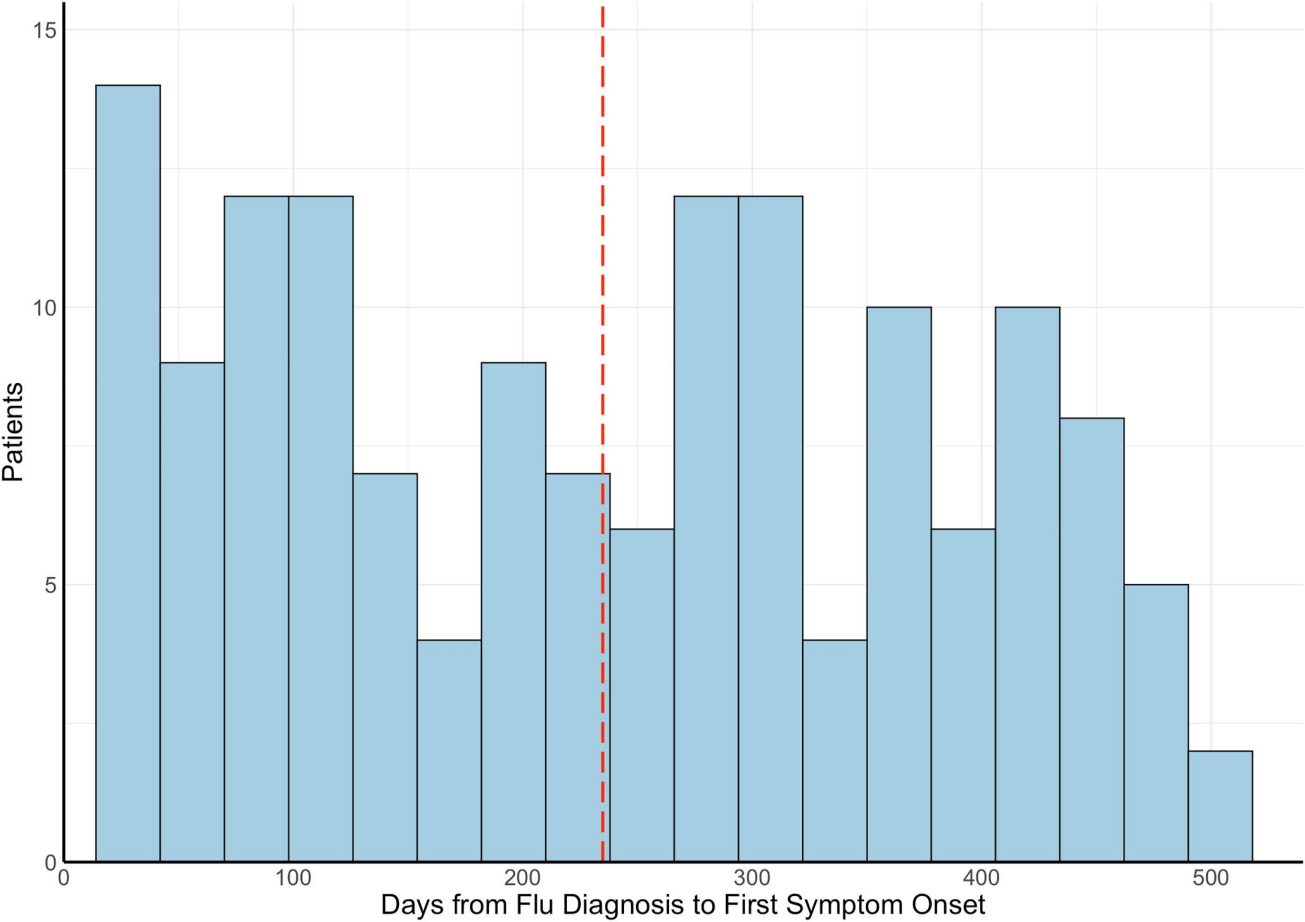

### 3.3 Neuro‐PASC associated drug prescription

The most commonly prescribed drugs for neuro‐PASC symptoms were antiemetics (15.2% of neuro‐PASC patients), followed by benzodiazepines (11.2%), anticonvulsants (9.6%), and antidepressants (8.8%; Figure 4a). COVID‐19 patients were prescribed these drugs significantly more frequently than flu patients after their respective diagnoses (*p* = 0.0060).

FIGURE 4(a) Most prescribed neuropsychiatric drugs post‐COVID‐19. (b) Patient hospitalization status versus number of prescribed drugs (*p* < 0.0001)

**Figure:**
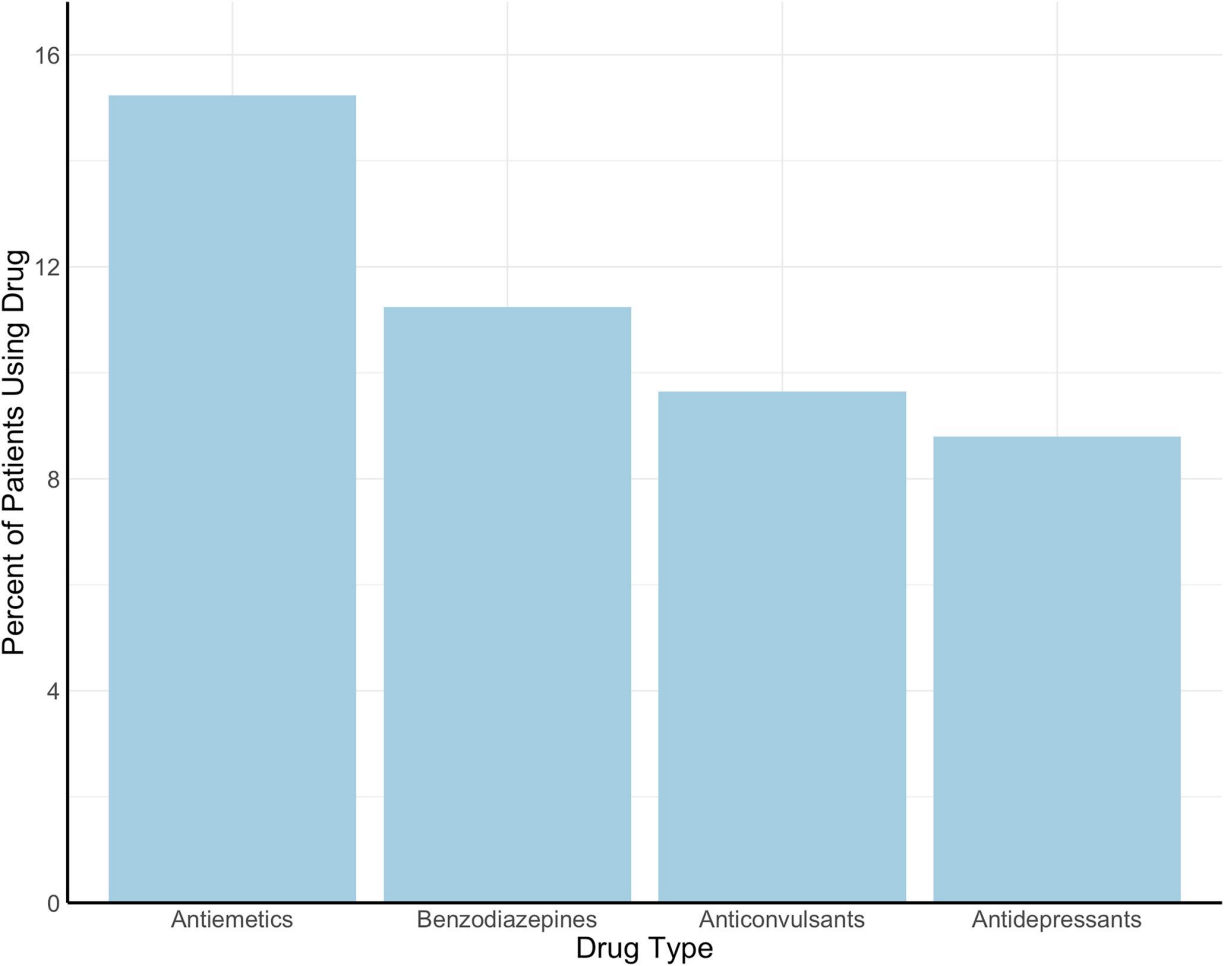

**Figure:**
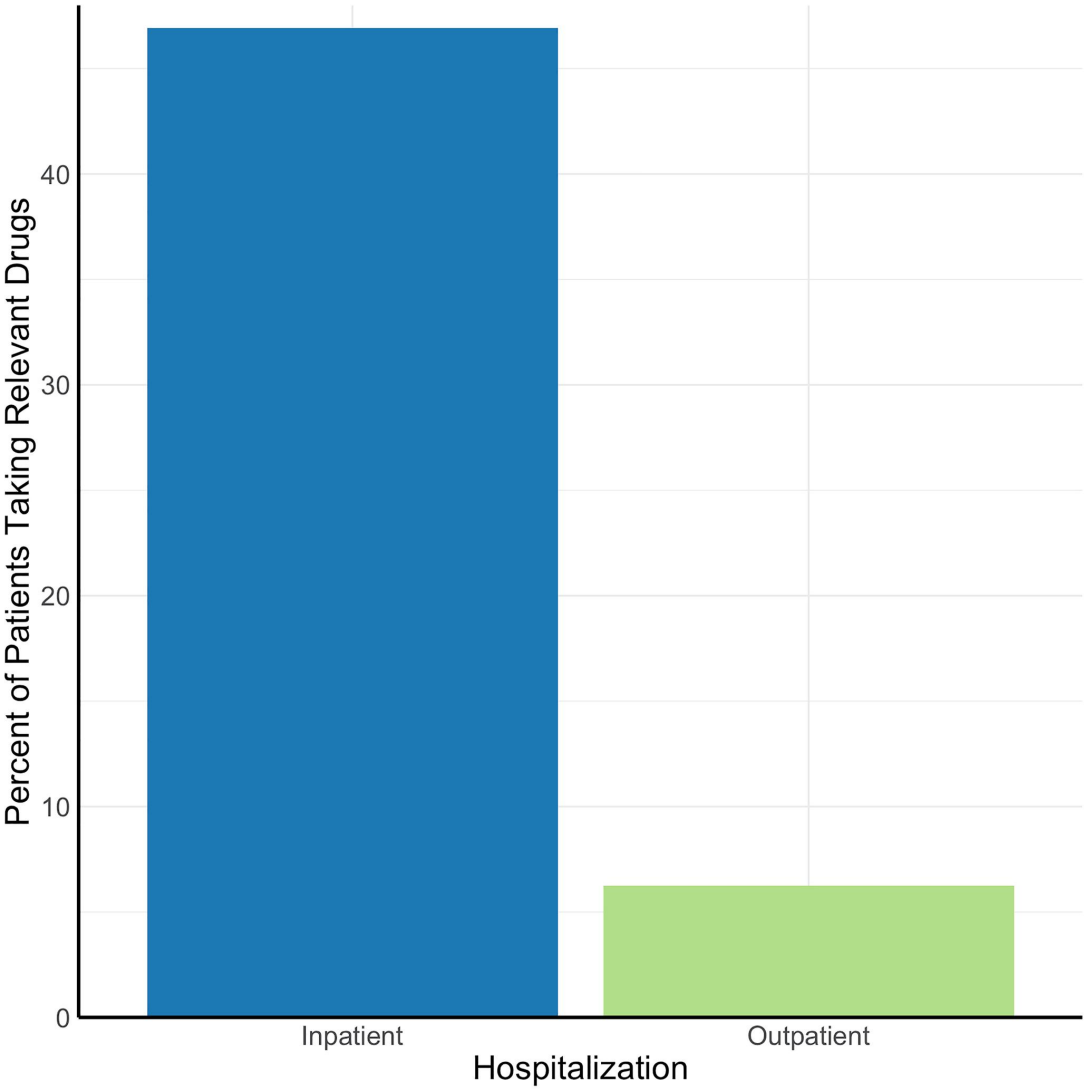

Antiemetics, anticonvulsants, and antidepressants, but not benzodiazepines, were prescribed more to females (*p* < 0.001). Anticonvulsants, antidepressants, and benzodiazepines, but not antiemetics, were prescribed more to older neuro‐PASC patients (*p* < 0.05). Drug prescriptions did not differ across racial groups (*p* > 0.05). Hospitalized patients were prescribed more of these drugs compared to non‐hospitalized patients (14.8% vs. 1.7%, *p* < 0.05; Figure 4b).

## 4 DISCUSSION

This study characterized new‐onset neurological and neuropsychiatric sequelae in COVID‐19 patients at least 2 weeks after COVID‐19 diagnosis using real‐world EHR data from a diverse population in the Bronx. Comparisons were made with COVID‐19 patients without neuro‐PASC as well as flu patients with neurological and neuropsychiatric sequelae from the same period and catchment area. Major findings are: (1) COVID‐19 survivors experienced new‐onset neuro‐PASC symptoms on average 138 days post COVID‐19 diagnosis, with hospitalized patients reporting earlier onset than non‐hospitalized patients; (2) the most common neuro‐PASC symptoms were anxiety disorders, depression, dizziness, fatigue, altered mental status, chronic headaches, and nausea; (3) the incidence of neuro‐PASC was significantly associated with female sex and older age, but not race, ethnicity, most comorbidities, or COVID‐19 disease severity; (4) the most commonly prescribed drugs for neuro‐PASC symptoms were antiemetics, benzodiazepines, anticonvulsants, and antidepressants, with more being prescribed to COVID‐19 patients who were hospitalized, female, and older, (5) neuro‐PASC COVID‐19 patients were significantly older, exhibited higher incidence of altered mental status, developed neuropsychiatric symptoms more quickly, and were prescribed associated drugs more often compared to flu patients, and (6) COVID‐19 patients with neuro‐PASC had more extreme laboratory values than flu patients. These findings underscore the likely neurotropism of SARS‐CoV‐2 infection, resulting in new significant neurological and neuropsychiatric sequelae among survivors regardless of COVID‐19 disease severity.

The most common neuro‐PASC symptoms identified were anxiety disorders, depression, dizziness, fatigue, altered mental status, chronic headaches, and nausea, in line with those reported previously (Carfì et al., 2020; Garrigues et al., 2020; Mao et al., 2020; Townsend et al., 2020). However, most prior studies were surveys and did not include detailed EHR data (Davis et al., 2021; Garrigues et al., 2020; Moghimi et al., 2021). Our analysis also excluded patients with pre‐existing neurological and neuropsychiatric symptoms and considered only new‐onset cases, whereas most previous studies have included individuals with such pre‐existing conditions (Carfì et al., 2020; Lamontagne et al., 2021). We further excluded hyperacute neuropsychiatric symptoms within the first 2 weeks after COVID‐19 diagnosis, whereas some prior investigations did not (Mao et al., 2020; Tenforde et al., 2020).

Onset of neuro‐PASC symptoms was earlier in COVID‐19 patients who required hospitalization. It is possible that severe COVID‐19 triggers sudden symptom onset, while mild COVID‐19 is associated with a more gradual neuro‐PASC symptom development. This finding suggests that screening for neuro‐PASC should start as early as possible, especially among those previously hospitalized for COVID‐19.

Female sex was associated with higher incidence of neuro‐PASC, even though women have lower risk of severe COVID‐19 outcomes than men (Jin et al., 2020). Sex differences in psychiatric symptom prevalence are well known, but the causes of these differences are not well understood and could be either biological or cultural (Riecher‐Rössler, 2017). Further investigation is consequently warranted in the context of COVID‐19. Older age was also associated with higher incidence of neuro‐PASC, consistent with previous findings that fatigue, anosmia, and other non‐neurological clinical symptoms of COVID‐19 infection are also significantly more prevalent in older individuals (Carvalho‐Schneider et al., 2021; Moghimi et al., 2021).

A major finding is that presence of neuro‐PASC symptoms was not associated with COVID‐19 severity, when the latter was measured by either hospitalization status, pulse oximetry values (indicating need for supplemental oxygen), oral temperature (indicating fever), or comorbidities linked with worse COVID‐19 outcomes. This result is further supported by several smaller studies (Henry et al., 2020; Lin et al., 2021; Mazza et al., 2020; Sahu et al., 2020) and by laboratory measures observed herein (in particular LDH, CRP, and D‐dimer), which were less extreme for individuals with neuro‐PASC, indicative of less severe COVID‐19 disease.

Taken together, these findings suggest that development of neuro‐PASC symptoms is not limited to hospitalized patients with severe COVID‐19 and supports prior results of multiple smaller investigations (Townsend et al., 2020; van den Borst et al., 2021; Garrigues et al., 2020). An important clinical implication is that screening for neuro‐PASC should be done for all individuals post SARS‐CoV‐2 infection, regardless of hospitalization.

Increased usage of neuropsychiatric drugs after discharge was associated with inpatient status/ICU hospitalization, female sex, and older age. The latter two associations are expected, as older and female patients also developed neuro‐PASC symptoms more frequently. Nevertheless, given the lack of association between COVID‐19 disease severity and the incidence of neuro‐PASC, the relationship between hospitalization and prescribed drugs warrants further investigation.

We compared neuropsychiatric sequelae of COVID‐19 patients with those of flu patients over the same period and in the same catchment area, enabled by the use of EHR. COVID‐19 patients with neuro‐PASC were markedly older, a difference that can be attributed to how COVID‐19 differentially affects the older population. Compared to flu patients, COVID‐19 patients with relevant sequelae had lower incidence of COPD/asthma, suggesting that COPD/asthma is a major risk factor for flu but less so for COVID‐19. Other comorbidities did not contribute to differences in incidence of new neuropsychiatric symptoms.

Neuropsychiatric symptoms in both cohorts were similar, except that COVID‐19 patients experienced higher incidence of altered mental status and trending higher incidence of fatigue. Symptoms in COVID‐19 patients also developed significantly sooner than in flu patients. AMS and fatigue are amongst the most reported COVID‐19 neuro‐PASC symptoms (Carfì et al., 2020; Garrigues et al., 2020; Mao et al., 2020; Townsend et al., 2020). Consequently, future studies should include quantitative measures of fatigue scores.

Drugs used to treat neuropsychiatric conditions were prescribed significantly more to COVID‐19 patients than to flu patients following their respective diagnoses, potentially indicating that neuro‐PASC is considerably more severe than sequelae of other respiratory conditions (Taquet et al., 2021). Compared to flu patients, COVID‐19 patients with neuro‐PASC had more extreme laboratory values in line with the typical range of laboratory abnormalities seen in COVID‐19 (Wiersinga et al., 2020).

This study has several limitations. Notably, it is a retrospective investigation relying on available EHR data. While EHR data offer numerous advantages, it is possible that some neuro‐PASC symptoms were not documented pre‐, during, or post‐COVID‐19 diagnosis. We considered only patients who returned to the hospital for care after COVID‐19 diagnosis and therefore an overall neuro‐PASC incidence rate could not be reported. For consistency, the control cohort included only flu patients diagnosed over the same time period (i.e., patients were likely to go through similar triage and testing). A disadvantage is that flu incidence during the COVID‐19 pandemic was lower than pre‐pandemic and likely underreported (Taquet et al., 2021). As a result, future work should focus on comparing COVID‐19 patient data with a broader flu cohort. Separately, the population examined contained a large proportion of Black and Hispanic patients, many of whom were of lower socioeconomic status, potentially rendering our findings not generalizable to other less diverse populations. Consequently, comparisons with other racial and ethnic groups are warranted. Additionally, our study did not quantify the degree of neuro‐PASC severity. Lastly, as with any retrospective study, there could be other unintended patient selection bias.

In conclusion, this study provides additional insights on neuro‐PASC associations with key clinical variables, which may be used to support at‐risk patients, develop effective interventions and screening methods, and understand the potential for future neurological and psychiatric sequelae related to COVID‐19.

## CONFLICT OF INTEREST

None.

## AUTHOR CONTRIBUTION

Andrei L. Iosifescu: Conceptualization, Data curation, Formal analysis, Investigation, Visualization, Writing — original draft, Writing — review & editing. Wouter S. Hoogenboom: Conceptualization, Investigation, Supervision, Writing — original draft, Writing — review & editing. Alexandra J. Buczek: Conceptualization, Writing — review & editing. Roman Fleysher: Data curation, Writing — review & editing. Tim Q. Duong: Conceptualization, Resources, Supervision, Writing — original draft, Writing — review & editing.

## Supporting information

Supporting Information S1

Supporting Information S2

## Data Availability

The data that support the findings of this study are available from the corresponding author upon reasonable request.

## References

[mpr1914-bib-0030] Anand, H. , Ende, V. , Singh, G. , Qureshi, I. , Duong, T. Q. , & Mehler, M. F. (2021). Nervous system‐systemic crosstalk in SARS‐CoV‐2/COVID‐19: A unique dyshomeostasis syndrome. Frontiers in Neuroscience, 15, 727060. 10.3389/fnins.2021.727060 34512253 PMC8430330

[mpr1914-bib-0001] Carfì, A. , Bernabei, R. , & Landi, F (2020). Persistent symptoms in patients after acute COVID‐19. JAMA, 324(6), 603–605. 10.1001/jama.2020.12603 32644129 PMC7349096

[mpr1914-bib-0002] Carvalho‐Schneider, C. , Laurent, E. , Lemaignen, A. , Beaufils, E. , Bourbao‐Tournois, C. , Laribi, S. , Flament, T. , Ferreira‐Maldent, N. , Bruyere, F. , Stefic, K. , Gaudy‐Graffin, C. , Grammatico‐Guillon, L. , & Bernard, L. (2021). Follow‐up of adults with noncritical COVID‐19 two months after symptom onset. Clinical Microbiology and Infection, 27(2), 258–263. 10.1016/j.cmi.2020.09.052 33031948 PMC7534895

[mpr1914-bib-0003] Collantes, M. E. V. , Espiritu, A. I. , Sy, M. C. C. , Anlacan, V. M. M. , & Jamora, R. D. G. (2021). Neurological manifestations in COVID‐19 infection: A systematic review and meta‐analysis. The Canadian Journal of Neurological Sciences, 48(1), 66–76. 10.1017/cjn.2020.146 32665054 PMC7492583

[mpr1914-bib-0004] Davis, H. E. , Assaf, G. S. , McCorkell, L. , Wei, H. , Low, R. J. , Re'em, Y. , Redfield, S. , Austin, J. P. , & Akrami, A. (2021). Characterizing long COVID in an international cohort: 7 months of symptoms and their impact. eClinicalMedicine, 38, 101019. 10.1016/j.eclinm.2021.101019 34308300 PMC8280690

[mpr1914-bib-0005] Garrigues, E. , Janvier, P. , Kherabi, Y. , Le Bot, A. , Hamon, A. , Gouze, H. , Doucet, L. , Berkani, S. , Oliosi, E. , Mallart, E. , Corre, F. , Zarrouk, V. , Moyer, J. D. , Galy, A. , Honsel, V. , Fantin, B. , & Nguyen, Y. (2020). Post‐discharge persistent symptoms and health‐related quality of life after hospitalization for COVID‐19. Journal of Infection, 81(6), e4–e6. 10.1016/j.jinf.2020.08.029 32853602 PMC7445491

[mpr1914-bib-0006] Graham, E. L. , Clark, J. R. , Orban, Z. S. , Lim, P. H. , Szymanski, A. L. , Taylor, C. , DiBiase, R. M. , Jia, D. T. , Balabanov, R. , Ho, S. U. , Batra, A. , Liotta, E. M. , & Koralnik, I. J. (2021). Persistent neurologic symptoms and cognitive dysfunction in non‐hospitalized Covid‐19 “long haulers”. Annals of Clinical and Translational Neurology, 8(5), 1073–1085. 10.1002/acn3.51350 33755344 PMC8108421

[mpr1914-bib-0007] Henry, B. M. , Aggarwal, G. , Wong, J. , Benoit, S. , Vikse, J. , Plebani, M. , & Lippi, G. (2020). Lactate dehydrogenase levels predict coronavirus disease 2019 (COVID‐19) severity and mortality: A pooled analysis. The American Journal of Emergency Medicine, 38(9), 1722–1726. 10.1016/j.ajem.2020.05.073 32738466 PMC7251362

[mpr1914-bib-0008] Hoogenboom, W. S. , Fleysher, R. , Soby, S , Mirhaji, P. , Mitchell, W. B. , Morrone, K. A. , Manwani, D. , & Duong, T. Q. (2021). Individuals with sickle cell disease and sickle cell trait demonstrate no increase in mortality or critical illness from COVID‐19—A fifteen hospital observational study in the Bronx, New York. Haematologica, 106(11), 3014. 10.3324/haematol.2021.279222 34348452 PMC8561299

[mpr1914-bib-0009] Hoogenboom, W. S. , Pham, A. , Anand, H. , Fleysher, R. , Buczek, A. , Soby, S. , Mirhaji, P. , Yee, J. , & Duong, T. Q. (2021). Clinical characteristics of the first and second COVID‐19 waves in the Bronx, New York: A retrospective cohort study. Lancet Reg Health Am, 3, 100041. 10.1016/j.lana.2021.100041 34423331 PMC8367084

[mpr1914-bib-0010] Hripcsak, G. , Duke, J. D. , Shah, N. H , Reich, C. G. , Huser, V. , Schuemie, M. J. , Suchard, M. A. , Park, R. W. , Wong, I. C. K. , Rijnbeek, P. R. , & Van Der Lei, J. (2015). Observational Health Data Sciences and Informatics (OHDSI): Opportunities for observational researchers. Studies in Health Technology and Informatics, 216, 574–578. 26262116 PMC4815923

[mpr1914-bib-0011] Jin, J. M. , Bai, P. , He, W. , Wu, F. , Liu, X. F. , Han, D. M. , Liu, S. , & Yang, J. K. (2020). Gender differences in patients with COVID‐19: Focus on severity and mortality. Frontiers in Public Health, 8, 152. 10.3389/fpubh.2020.00152 32411652 PMC7201103

[mpr1914-bib-0012] Lamontagne, S. J. , Winters, M. F. , Pizzagalli, D. A. , & Olmstead, M. C. (2021). Post‐acute sequelae of COVID‐19: Evidence of mood & cognitive impairment. Brain, Behavior, and Immunity ‐ Health, 17, 100347. 10.1016/j.bbih.2021.100347 PMC8437695 34549199

[mpr1914-bib-0013] Lin, J. , Yan, H. , Chen, H. , He, C. , Lin, C. , He, H. , Zhang, S. , Shi, S. , & Lin, K. (2021). COVID‐19 and coagulation dysfunction in adults: A systematic review and meta‐analysis. Journal of Medical Virology, 93(2), 934–944. 10.1002/jmv.26346 32706426 PMC7405098

[mpr1914-bib-0014] Lu, J. Q. , Lu, J. Y. , Wang, W. , Liu, Y. , Buczek, A. , Fleysher, R. , Hoogenboom, W. S. , Zhu, W. , Hou, W. , Rodriguez, C. J. , & Duong, T. Q. (2022). Clinical predictors of acute cardiac injury and normalization of troponin after hospital discharge from COVID‐19. EBioMedicine, 76, 103821. 10.1016/j.ebiom.2022.103821 35144887 PMC8819639

[mpr1914-bib-0031] Lu, J. Y. , Buczek, A. , Fleysher, R. , Hoogenboom, W. S. , Hou, W. , Rodriguez, C. J. , Fisher, M. C. , & Duong, T. Q. (2021). Outcomes of hospitalized patients with COVID‐19 with acute kidney injury and acute cardiac injury. Frontiers in cardiovascular medicine., 8, 798897. 10.3389/fcvm.2021.798897 35242818 PMC8886161

[mpr1914-bib-0015] Ludvigsson, J. F. (2021). Case report and systematic review suggest that children may experience similar long‐term effects to adults after clinical COVID‐19. Acta Paediatrica, 110(3), 914–921. 10.1111/apa.15673 33205450 PMC7753397

[mpr1914-bib-0016] Mao, L. , Jin, H. , Wang, M. , Hu, Y. , Chen, S. , He, Q. , Chang, J. , Hong, C. , Zhou, Y. , Wang, D. , Miao, X. , Li, Y. , & Hu, B. (2020). Neurologic manifestations of hospitalized patients with coronavirus disease 2019 in Wuhan, China. JAMA Neurology, 77(6), 683–690. 10.1001/jamaneurol.2020.1127 32275288 PMC7149362

[mpr1914-bib-0017] Mazza, M. G. , De Lorenzo, R. , Conte, C. , Poletti, S. , Vai, B. , Bollettini, I. , Melloni, E. M. T. , Furlan, R. , Ciceri, F. , Rovere‐Querini, P. , & Benedetti, F. (2020). Anxiety and depression in COVID‐19 survivors: Role of inflammatory and clinical predictors. Brain, Behavior, and Immunity, 89, 594–600. 10.1016/j.bbi.2020.07.037 32738287 PMC7390748

[mpr1914-bib-0018] Moghimi, N. , Di Napoli, M. , Biller, J. , Siegler, J. E. , Shekhar, R. , McCullough, L. D. , Harkins, M. S. , Hong, E. , Alaouieh, D. A. , Mansueto, G. , & Divani, A. A. (2021). The neurological manifestations of post‐acute sequelae of SARS‐CoV‐2 infection. Current Neurology and Neuroscience Reports, 21(9), 44. 10.1007/s11910-021-01130-1 34181102 PMC8237541

[mpr1914-bib-0019] Riecher‐Rössler, A. (2017). Sex and gender differences in mental disorders. The Lancet Psychiatry, 4(1), 8–9. 10.1016/S2215-0366(16)30348-0 27856397

[mpr1914-bib-0020] Rubin, R. (2020). As their numbers grow, COVID‐19 "Long Haulers" stump experts. JAMA, 324(14), 1381–1383. 10.1001/jama.2020.17709 32965460

[mpr1914-bib-0021] Sahu, B. R. , Kampa, R. K. , Padhi, A. , & Panda, A. K. (2020). C‐reactive protein: A promising biomarker for poor prognosis in COVID‐19 infection. Clinica Chimica Acta, 509, 91–94. 10.1016/j.cca.2020.06.013 PMC7274122 32511972

[mpr1914-bib-0022] Taquet, M. , Geddes, J. R. , Husain, M. , Luciano, S. , & Harrison, P. J. (2021). 6‐month neurological and psychiatric outcomes in 236 379 survivors of COVID‐19: a retrospective cohort study using electronic health records. The Lancet Psychiatry, 8(5), 416–427. 10.1016/S2215-0366(21)00084-5 33836148 PMC8023694

[mpr1914-bib-0023] Tenforde, M. W. , Kim, S. S. , Lindsell, C. J. , Billig Rose, E. , Shapiro, N. I. , Files, D. C. , Gibbs, K. W. , Erickson, H. L. , Steingrub, J. S. , Smithline, H. A. , Gong, M. N. , Aboodi, M. S. , Exline, M. C. , Henning, D. J. , Wilson, J. G. , Khan, A. , Qadir, N. , Brown, S. M. , Peltan, I. D. , …, & Wu, M. J. (2020). Symptom duration and risk factors for delayed return to usual health among outpatients with COVID‐19 in a multistate health care systems network—United States, March–June 2020. MMWR Morbidity and Mortality Weekly Report, 69(30), 993–998. 10.15585/mmwr.mm6930e1 32730238 PMC7392393

[mpr1914-bib-0024] Townsend, L. , Dyer, A. H. , Jones, K. , Dunne, J. , Mooney, A. , Gaffney, F. , O'Connor, L. , Leavy, D. , O'Brien, K. , Dowds, J. , Sugrue, J. A. , Hopkins, D. , Martin‐Loeches, I. , Ni Cheallaigh, C. , Nadarajan, P. , McLaughlin, A. M. , Bourke, N. M. , Bergin, C. , O'Farrelly, C. , …, Conlon, N. (2020). Persistent fatigue following SARS‐CoV‐2 infection is common and independent of severity of initial infection. PLoS One, 15(11), e0240784. 10.1371/journal.pone.0240784 33166287 PMC7652254

[mpr1914-bib-0025] van den Borst, B. , Peters, J. B. , Brink, M. , Schoon, Y. , Bleeker‐Rovers, C. P. , Schers, H. , van Hees, H. W. H. , van Helvoort, H. , van den Boogaard, M. , van der Hoeven, H. , Reijers, M. H. , Prokop, M. , Vercoulen, J. , & van den Heuvel, M. (2021). Comprehensive health assessment 3 months after recovery from acute coronavirus disease 2019 (COVID‐19). Clinical Infectious Diseases, 73(5), e1089–e1098. 10.1093/cid/ciaa1750 33220049 PMC7717214

[mpr1914-bib-0026] Wadhera, R. K. , Wadhera, P. , Gaba, P. , Figueroa, J. F. , Joynt Maddox, K. E. , Yeh, R. W. , & Shen, C. (2020). Variation in COVID‐19 hospitalizations and deaths across New York City boroughs. JAMA, 323(21), 2192–2195. 10.1001/jama.2020.7197 32347898 PMC7191469

[mpr1914-bib-0027] Wang, F. , Kream, R. M. , & Stefano, G. B. (2020). Long‐term respiratory and neurological sequelae of COVID‐19. Medical Science Monitor, 26, e928996. 10.12659/MSM.928996 33177481 PMC7643287

[mpr1914-bib-0028] Wiersinga, W. J. , Rhodes, A. , Cheng, A. C. , Peacock, S. J. , & Prescott, H. C. (2020). Pathophysiology, transmission, diagnosis, and treatment of coronavirus disease 2019 (COVID‐19): A review. JAMA, 324(8), 782–793. 10.1001/jama.2020.12839 32648899

[mpr1914-bib-0029] Zhao, Y. M. , Shang, Y. M. , Song, W. B. , Li, Q. q. , Xie, H. , Xu, Q. f. , Jia, J. l. , Li, L. m. , Mao, H. l. , Zhou, X. m. , Luo, H. , Gao, Y. f. , & Xu, A. g. (2020). Follow‐up study of the pulmonary function and related physiological characteristics of COVID‐19 survivors three months after recovery. eClinicalMedicine, 25, 100463. 10.1016/j.eclinm.2020.100463 32838236 PMC7361108

