# Supplementary material for: New‐onset and persistent neurological and psychiatric sequelae of COVID‐19 compared to influenza: A retrospective cohort study in a large New York City healthcare network
Source: Int J Methods Psychiatr Res. 2022 Jun 15;31(3):e1914. doi: 10.1002/mpr.1914 (PMC9349863; doi:10.1002/mpr.1914)
Supplement: Supplementary file 1 — Supporting Information S1 [file MPR-31-e1914-s002.docx]

**Supplementary Materials**

**New-onset and persistent neurological and neuropsychiatric sequelae of COVID-19 and influenza in a New York City healthcare network**

Andrei L. Iosifescu, Wouter S. Hoogenboom, Alexandra J. Buczek, Roman Fleysher, Tim Q. Duong

**Supplementary Table 1**

*OMOP Concept IDs Used to Define Neuro-PASC Symptoms*

| **Symptom** | **OMOP Concept IDs** |
| --- | --- |
| Altered Mental Status | 4162499, 436222 |
| Altered Smell | 43530714, 4307095, 444223, 4185711, 4168062, 4224339 |
| Altered Taste | 43530714, 436235, 4240713, 4266190, 4289517 |
| Anxiety and PTSD | 36684319, 4199892, 4338031, 381537, 37117155, 442077, 4263429, 4115221, 4214746, 4113821, 4322025, 441542, 443414, 436676, 440083 |
| Ataxia | 4105349, 42535411, 43531622, 435242, 40483772, 437584 |
| Brain Hemorrhage | 44782730, 4045744, 376713, 42872427 |
| Brain Lesion | 4173938, 4200516, 37109019 |
| Delirium | 379779, 373995 |
| Depression | 4019705, 35622934, 4299785, 441534, 438406, 435220, 4154309, 439254, 4323418, 4025677, 35624748, 4161569, 35624743, 4141454, 4250023, 4263748, 4049623, 37111697, 433991, 42872411, 4148630, 4195572, 4269493, 43531624, 4282096, 379784, 4176002, 4332994, 36717092, 4282316, 4307111, 42872722, 4095285, 4114950, 4239471, 4336957, 4151170, 4314692, 36713698, 4149321, 4149320, 40546087, 4282096, 4287238, 4181807, 434911, 4220023, 4037669, 4324959, 37018656, 432883, 4094358, 438998, 4224940, 4338031, 440698, 432285, 438727, 4152280, 440383 |
| Dizziness | 4180463, 4250121, 433316, 4012243, 4223938 |
| Fatigue | 4202045, 4063119, 432738, 45772721, 439926, 4223659 |
| Headache and Migraine | 762083, 762084, 762152, 42535410, 377546, 36716800, 4318560, 377545, 40482953, 377853, 43530648, 374922, 4047909, 43530641, 44782681, 380093, 378145, 44782806, 373463, 46270383, 376104, 376382, 373755, 4283532, 375527, 4134454, 4162234, 381278, 4012515, 378253, 760968, 43530652, 4314161, 44782470, 443616, 372886, 762769, 378735, 4105620, 4141827, 433763, 443615, 4317966, 376394, 381549, 4294246, 379806, 318736 |
| Memory Loss | 4141586, 4084412, 4145069, 4304008 |
| Mood Disorder | 4298317, 37117214, 4328217, 4295956, 436079, 37109941, 44784632, 373176, 4269143, 444100 |
| Nausea | 44783646, 4104544, 45757468, 27674, 31967 |
| Psychotic Disorder | 436952, 436073 |
| Seizure and Epilepsy | 762834, 43530665, 762957, 4236312, 762812, 374168, 4196708, 377101, 4195615, 4233724, 4183856, 4078333, 40481467, 4029498, 4106574, 4194232, 377091, 37016270, 4101747, 40483585, 4046218, 439654, 40480055, 46270367, 43530734, 4044082, 45757050, 376680, 4216711, 374924, 4274575, 374915, 4310999, 4326435, 4102345, 40483317, 380378 |
| Stroke / Cerebrovascular Accident | 761790, 762340, 44782781, 761791, 762345, 43531605, 43530665, 761798, 44782753, 440426, 4046363, 4111710, 4211509, 4159140, 36716999, 4189462, 4310996, 4153352, 36684840, 37395575, 37395576, 4110196, 42535412, 4112022, 42535508, 42535110, 761785, 42535111, 42535112, 42535113, 42535114, 40482301, 42535148, 42535461, 36684470, 42539262, 4045734, 37395574, 765568, 381316, 42539256, 381591, 4121341, 40484120, 374060, 443454, 4319331, 4077086, 4046362 |
| Tinnitus | 36685115, 36685258, 36685187, 4339489, 764634, 764632, 381864, 45763549, 4320814, 377575 |
